# Black esophagus: a case report

**DOI:** 10.1186/1757-1626-1-367

**Published:** 2008-12-02

**Authors:** Maha M Maher, Mahmoud I Nassar

**Affiliations:** 1 Gastroenterology Department, Mansoura University, Internal Medicine Specialty Hospital, Mansoura, Egypt; 2 Department of Pathology, Assiut University, Assiut, Egypt

## Abstract

The black esophagus is a rare observation during upper endoscopy. We describe a case of a male with chronic obstructive pulmonary disease, hypertension, diabetes, acute renal failure and in septic state, who developed a black esophagus after hypotensive episodes. The clinical, endoscopic and histopathological characteristics are presented.

## Background

The "black esophagus" is an extremely rare endoscopic finding since its first description by Golden berg in 1990 [1]. Although black discoloration can be caused by malignant melanoma, melanosis, pseudomelanosis, acanthosis nigrigans, or coal dust deposition, the term "black esophagus" most often refers to acute esophageal necrosis "AEN" [2]. AEN is frequently associated with severe clinical conditions such as hypovolemic shock, septic shock, hyperglycemia, hypothermia, and liver disease, with impairment of the hemodynamic equilibrium of the patient[3]. What is known about black esophagus is based primarily on case reports and small case series. In the present report we describe the clinical, endoscopic and histopathological characteristics of a patient with a diagnosis of ANE.

## Case presentation

A 63 – years old man, with diabetes mellitus type II, arterial hypertension and chronic obstructive pulmonary disease was admitted to internal medicine department suffering from dyspnea, cough and expectoration. Initial physical examination revealed a patient with poor general condition, normotensive, pulse 92 b.p.m, temperature was 37.8°C. Patient suddenly desaturated ; O_2 _saturation 78%, blood pressure decreased to 80/40, patient transferred to ICU, connected to mechanical ventilator. laboratory investigation revealed a state of acute renal failure. At this point a significant amount of coffee ground material (about 500 CC) was drained through nasogastric tube. Hemoglobin 9.3 g/dL, leukocytes 23 cells/mm 3, platelets 201 U/L, sodium 141 mEqu/L, potassium 4 mEqu/L, Glu 11.5 mmol/L, BUN 16.2, creatinine 254, urine culture revealed pseudomonas aereogenosus.

**Figure 1:**
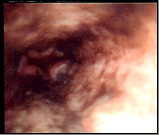
Black esophageal mucosa of lower 2/3.

**Figure 2:**
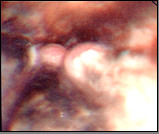
Two small nodules at cardioesophageal junction.

Upper GIT endoscopy showed : a blackened and friable esophageal mucosa mostly at lower 2/3 with gradual fading of darkness in upper 1/3 (figure 1), two small nodules presented at cardioesophageal junction (figure 2), biopsies taken from esophageal mucosa and nodules. Superficial gastric erosions with stigmata of recent bleeding, duodenum appears normal.

**Figure 3:**
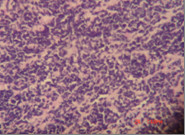
Small rounded cell large nucleated with scares or thin rim of cytoplasm tendency to be arranged in a rosette manner.

**Figure 4:**
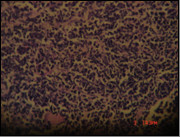
**Frequent necrotic cells, apoptotic changes with focal necrotic areas.** Small blood vessels with frequent hyalenosis.

Histopathology: Sections show fragments of esophageal squamous epithelium and antral mucosal tissue, they feature intraepithelial acute inflammatory cellular reaction, edema of the antral mucosa. It contains fragments of neoplastic growth formed of small separate rounded cell infiltrating the esophageal subepithelial and the antral mucosa. It is formed of large nucleated cells (mostly rounded) with scares or thin rim of cytoplasm. It shows no tendency to form acini, there is a tendency to be arranged in a rosette like manner (Figure 3), it features frequent individual cell necrotic and apoptotic changes associated with focal necrotic areas, small blood vessels with frequent hyalenosis are seen (Figure 4).

The patient was treated with proton pump inhibitor (initially 80 mg omeprazole intravenously). Plan for abdominal CT with contrast but acute renal failure interfere to do it, arrangement for MRI however patient condition deteriorated through the following days and died.

## Discussion

Acute esophageal necrosis is a rare condition reported in the literature. In endoscopic studies the frequency ranged from 0.01% to 0.2% [4]. Men are markedly preponderant (88.5%) [5], men may be more predisposed to esophageal ischemia because at any age, they are more likely to have atherosclerotic vascular disease than woman [6]. Elderly and immunodepressed patients have a higher risk of developing this condition [7]. Haviv et al [8] have proposed that, because elderly patients have comorbid conditions that predispose them to a low hemodynamic flow state, they are at greater risk for black esophagus.

The etiology of AEN undefined and is probably multifactorial. Most investigators suggest an ischemic origin of esophagus [9], this observation seems implausible since the esophagus has a rich segmental and intramural blood supply. The preferential location of the lesion is the distal segment of the esophagus, which has been shown to be less vascularized in anatomical studies [3]. In addition this disease usually develops in states of low flow [4,5,8]. The next most common comorbidity was coexisting cancer, patients with cancer may be hypercoaguable and predisposed to thrombosis with subsequent ischemia of the esophageal mucosa [2]. Other significant comorbodities include lung disease (COPD, lung cancer) [10], diabetes mellitus, [11] renal insufficiency, [12] prolonged hypotension or sepsis which cause selective arterial or venous thrombosis of the esophagus [13]. In our case report ; age, debility due to diabetes, COPD, cancer, acute renal failure, sepsis and recurrent shock state seem to have played an important role in the selective esophageal ischemia. Also, endoscopic findings showed two small nodules at cardioesophageal junction ; the differential diagnosis of thin neoplastic growth should include ; gastric lymphoma (mucosal associated lymphocitic tumour), poorly differentiated adenocarcinomatous growth, neuroendocrinal carcinoma of the G I T, metastatic neuroplastoma (most unlikely because of the age of the patient). However this differential should be clarified and accompanied with immuno-stainig phenotyping studies. Unfortunately because of the small sized biopsy and the death of the patient this study was not possible. The main clinical presentation of black esophagus is hematemesis, but epigastric pain and dysphagia were other reported symptoms [2]. Management of this condition is based on the treatment of the co- morbid condition, appropriate hydration, and administration of proton pump inhibitor [4]. The prognosis of AEN patients is variable and depends on the severity of the associated clinical condition [3].

## Conclusion

black esophagus is a rare endoscopic finding reported in severely compromised elderly patients. The probable triggers are hypotension and sepsis. The prognosis of patients with AEN seems to be related to the underlying medical conditions and not to esophageal complications.

## Consent

"Written informed consent was obtained from the patient relative for publication of this case report and accompanying images. A copy of the written consent is available for review by the Editor-in-Chief of this journal"

## Competing interests

The authors declare that they have no competing interests.

## Authors' contributions

MM was responsible for upper endoscopy done and biopsies taken from the patient, endoscopic figures shown in manuscript. Also, design, acquisition of data, analysis and interpretation of data; have been involved in drafting the manuscript and revising it critically for important intellectual content; and have given final approval of the version to be published. MN was responsible for pathological studies of biopsies, gave his comments and interpretation of data.
